# The health and economic benefits of the global programme to eliminate lymphatic filariasis (2000–2014)

**DOI:** 10.1186/s40249-016-0147-4

**Published:** 2016-05-24

**Authors:** Hugo C. Turner, Alison A. Bettis, Brian K. Chu, Deborah A. McFarland, Pamela J. Hooper, Eric A. Ottesen, Mark H. Bradley

**Affiliations:** London Centre for Neglected Tropical Disease Research, London, UK; Department of Infectious Disease Epidemiology, School of Public Health, Faculty of Medicine, St Marys Campus, Imperial College London, Norfolk Place, London, W2 1PG UK; Neglected Tropical Diseases Support Center, Task Force for Global Health, Decatur, GA USA; Rollins School of Public Health, Emory University, Atlanta, GA USA; Global Health Programs, GlaxoSmithKline, London, UK

**Keywords:** Lymphatic filariasis, DALYs averted, Health impact, Economic impact, GPELF, Programme evaluation

## Abstract

**Background:**

Lymphatic filariasis (LF), also known as elephantiasis, is a neglected tropical disease (NTD) targeted for elimination through a Global Programme to Eliminate LF (GPELF). Between 2000 and 2014, the GPELF has delivered 5.6 billion treatments to over 763 million people. Updating the estimated health and economic benefits of this significant achievement is important in justifying the resources and investment needed for eliminating LF.

**Method:**

We combined previously established models to estimate the number of clinical manifestations and disability-adjusted life years (DALYs) averted from three benefit cohorts (those protected from acquiring infection, those with subclinical morbidity prevented from progressing and those with clinical disease alleviated). The economic savings associated with this disease prevention was then analysed in the context of prevented medical expenses incurred by LF clinical patients, potential income loss through lost-labour, and prevented costs to the health system to care for affected individuals. The indirect cost estimates were calculated using the human capital approach. A combination of four wage sources was used to estimate the fair market value of time for an agricultural worker with LF infection (to ensure a conservative estimate, the lowest wage value was used).

**Results:**

We projected that due to the first 15 years of the GPELF 36 million clinical cases and 175 (116–250) million DALYs will potentially be averted. It was estimated that due to this notable health impact, US$100.5 billion will potentially be saved over the lifetimes of the benefit cohorts. This total amount results from summing the medical expenses incurred by LF patients (US$3 billion), potential income loss (US$94 billion), and costs to the health system (US$3.5 billion) that were projected to be prevented. The results were subjected to sensitivity analysis and were most sensitive to the assumed percentage of work hours lost for those suffering from chronic disease (changing the total economic benefit between US$69.30–150.7 billion).

**Conclusions:**

Despite the limitations of any such analysis, this study identifies substantial health and economic benefits that have resulted from the first 15 years of the GPELF, and it highlights the value and importance of continued investment in the GPELF.

**Electronic supplementary material:**

The online version of this article (doi:10.1186/s40249-016-0147-4) contains supplementary material, which is available to authorized users.

## Multilingual abstracts

Please see Additional file 1 for translations of the abstract into the five official working languages of the United Nations.

## Background

Lymphatic filariasis (LF), also known as elephantiasis, is a neglected tropical disease (NTD) now targeted for elimination through the Global Programme to Eliminate LF (GPELF). When this Programme began in 2000, there were 1.3 billion people in 73 endemic countries at-risk of LF. It was estimated that approximately 120 million people were infected with LF, of which 40 million were suffering from overt clinical disease – manifested as painful severe swelling due to lymphedema (an accumulation of lymphatic fluid generally in the limbs) and hydrocele (fluid accumulation in the scrotal sac) [1, 2].

As a public health problem, LF is treated with preventative chemotherapy (Table 1) using a combination of either albendazole and ivermectin (in areas co-endemic with onchocerciasis) or albendazole and diethylcarbamazine (DEC) elsewhere. To achieve its elimination goals, the GPELF guides endemic countries in implementing annual, single-dose mass drug administration (MDA) against LF using either of these two drug regimens. The MDA strategy is unique in targeting all people of all ages in the endemic population who are eligible to take these safe and effective medicines (Table 1). This strategy has been shown to be feasible, inexpensive and cost-effective [2, 3], principally because the drugs used are donated by pharmaceutical partners.

**Table 1:** Glossary

**Acute disease/acute adenolymphangitis (ADL):** defined by symptoms of pain, tenderness, local swelling, and warmth in the groin or limbs with constitutional symptoms (such as fever, nausea, and vomiting). **Benefit Cohort 1:** individuals protected from acquiring infection. **Benefit Cohort 2:** individuals with existing subclinical morbidity protected from progression to clinical disease. **Benefit Cohort 3:** individuals with existing clinical morbidity for whom clinical morbidity was alleviated. **Chronic disease:** disease that is persistent (here limited specifically to hydrocele and lymphedema) **Clinical disease:** a disease that has recognizable clinical signs and symptoms (i.e. any acute or chronic cases). **Direct costs:** costs associated with medical resource utilization, which include the cost borne by the patients (for the medication and transport etc.) and the health system (i.e. personnel and capital resources etc.). **Discounting/Discount rate:** the process for adjusting future costs and outcomes to a “present value” to reflect the fact that a dollar is worth more today than it would be worth tomorrow. The discount rate determines the strength for the time preference. **Human capital approach:** human-capital method takes the patient’s perspective and counts any hour not worked as an hour lost. By contrast, the friction-cost method takes the employer’s perspective, and only counts as lost those hours not worked until another employee takes over the patient’s work [86]. **Hydrocele**: fluid accumulation in the scrotal sac. **Indirect costs:** expenses incurred from the cessation or reduction of work productivity as a result of the morbidity and mortality associated with a given disease. **Lymphedema:** an accumulation of lymphatic fluid generally in the limbs **Mass drug administration (MDA)/preventative chemotherapy:** administration of drugs to whole target population.	

Over the first eight operational years of the GPELF (2000–2007), more than 1.9 billion MDA treatments were administered to approximately 570 million individuals in 48 countries. This notable programmatic achievement resulted in a significant impact on the health of endemic populations [2]. A previous analysis by Chu et al. [4] estimated that due to GPELF activities between 2000 and 2007, over US$23 billion of economic benefits would be accrued by individuals and the health systems in MDA-treated areas (over the lifetime of those treated).

**Fig. 1:**
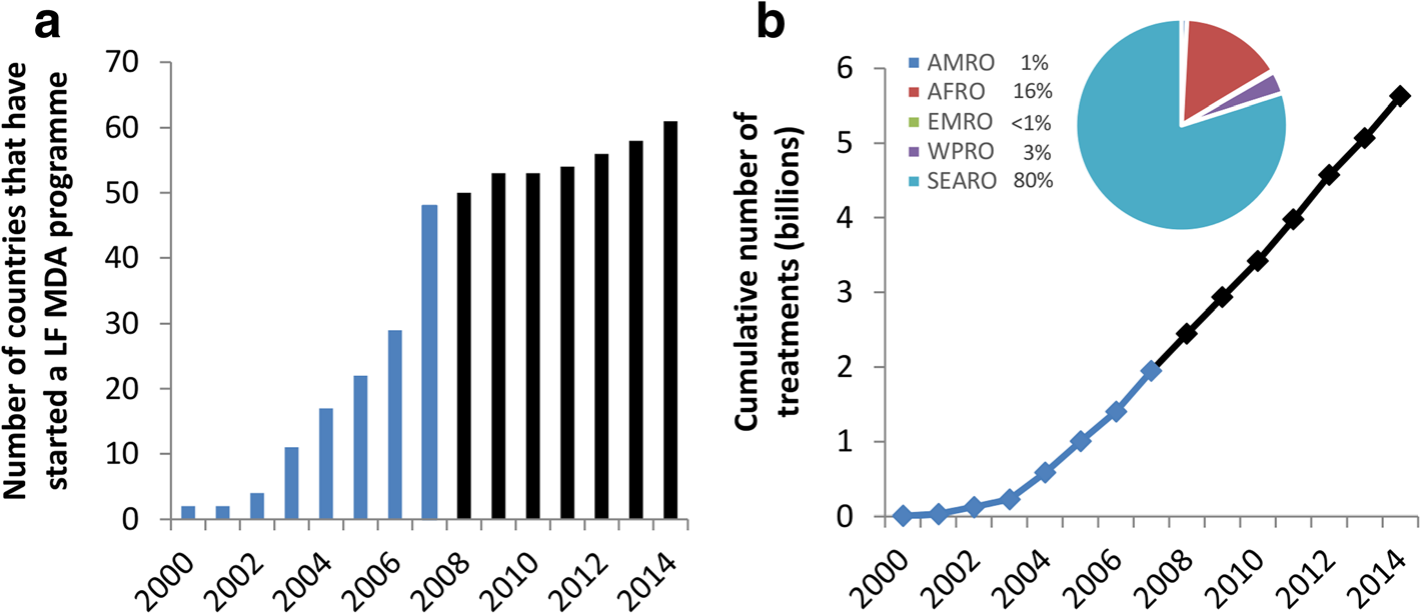
The number of countries that have started a LF MDA programme (**a**) and the cumulative number of treatments (**b**) provided by the GPELF over time. Data from the PCT databank [5]. Values in black indicate data provided after the timeframe of the previous analyses (2000–2007) [2, 4]. Insert in panel **b** illustrates the proportion of the cumulative number of treatments (2000–2014) in each of the different WHO regions (*AMRO* Region of the Americas, *AFRO* African Region, *EMRO* Eastern Mediterranean Region, *WPRO* Western Pacific Region, *SEARO* South-East Asia Region)

Since 2007, more countries have started MDA and others have expanded their treatment coverage (Fig. 1). This was made possible largely by new resource commitments for programme implementation from bilateral donors (especially DFID and USAID), the Bill & Melinda Gates Foundation, and other non-governmental development organizations (NGDOs) – funds that were needed to deliver the increased drug contributions from industry partners (initially GSK and Merck & Co, and more recently Eisai).

Assessment of the economic and health impact of the GPELF was first made after 8 years of programme activity [2, 4], but now given both the expansion of the programme during its subsequent seven years and the new strategies available for modelling its impact, the present manuscript aims to provide updated estimates of the health and economic benefits of the GPELF from 2000 to 2014.

## Methods

### GPELF – numbers at risk, and numbers treated

As seen in Table 2, since 2007, the programme has expanded to a further 13 countries and delivered over 3.68 billion more treatments (Table 2). Therefore, the number of people at-risk of infection and the numbers treated that were reported by Chu et al. [4] could be updated from the World Health Organisation (WHO) PCT databank [5] and the 2014 GPELF progress report [6]. The numbers of people at-risk of infection were estimated from data resulting from new mapping activities, and the numbers and populations treated, from countries’ annual reports to WHO and the drug donation programmes (summarized in the PCT databank [5]).

**Table 2:** GPELF MDA treatments (2000–2014)

WHO region	GPELF countries (2000–2014)^a^	Pre-control number at-risk of infection (*millions*)^b^	Minimum number treated 2000–2014 (*millions*)^c^
AMRO	Brazil, Dominican Republic, Guyana, Haiti.	14	10
AFRO	Benin, Burkina Faso, Cameroon, **Central African Republic,** Comoros, **Congo, Côte d’Ivoire, Democratic Republic of Congo, Ethiopia,** Ghana, **Guinea, Guinea-Bissau,** Kenya, **Liberia,** Madagascar, **Malawi,** Mali, **Mozambique,** Niger, Nigeria, Senegal, Sierra Leone, Tanzania (incl. Zanzibar), Togo, Uganda.	425	191
EMRO	Egypt, **Sudan** Yemen.	23	3
WPRO	American Samoa, **Brunei Darussalam**, Cambodia, Cook Islands, Fed. States of Micronesia, Fiji, French Polynesia, Kiribati, **Lao PDR**, Marshall Islands, Malaysia, Niue, Papua New Guinea, Philippines, Samoa, Tonga, Tuvalu, Vanuatu, Vietnam, Wallis and Futuna.	45	24
SEARO	Bangladesh, India, Indonesia, Maldives, Myanmar, Nepal, Sri Lanka, Thailand, Timor-Leste.	902	536
All Regions	**61 countries in total** ^d^	**1409**	**763**

*AMRO* Region of the Americas, *AFRO* African Region, *EMRO* Eastern Mediterranean Region, *WPRO* Western Pacific Region, *SEARO* South-East Asia Region

^a^Countries that started since 2007 are indicated in bold

^b^Data taken from [5, 6]

^c^A conservative approach was taken and the number of uniquely treated individuals in any one country was assumed to be the maximum number of individuals treated in any single MDA for each country

^d^ Palau has passed the TAS survey but never started MDA so is not included

### Epidemiological model and assumptions

**Fig. 2:**
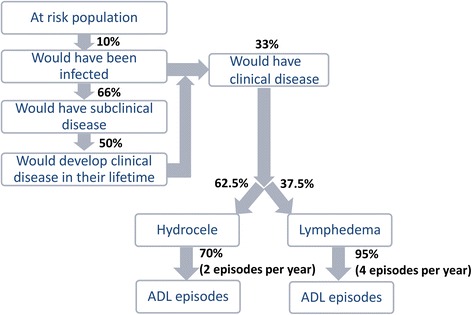
Baseline model assumptions. Assumptions based on [2, 4]. The sources for the parameters are outlined in Table 3

Based on previous analyses [2, 4], the following key assumptions were made (Fig. 2 and Table 3):

- Before control, 10 % of the at-risk population would be infected with LF, and this ratio remains constant in the absence of MDA [2].
- One-third of those with LF infections would have clinical disease (3.33 % of the total at-risk population).
- The other two-thirds of individuals infected with LF actually have *subclinical* morbidity [1]; half of these cases would progress to overt clinical disease in their lifetimes [2].
- Cases of clinical disease occur in the following proportion: 62.5 % hydrocele, 37.5 % lymphedema [7]. It was assumed that the relative frequency of the clinical disease presentations remains stable among those infected individuals.

**Table 3:** Baseline model parameters (based on [2, 4])

Parameter	Hydrocele average estimate	Lymphedema average estimate	Source
*Acute Disease*			
Percentage of clinical patients who experience ADL episodes per year	70 %	95 %	[8, 10–17]
Frequency of ADL episodes for clinical patients (in absence of MDA)	2 per year	4 per year	[8, 10–17]
Average duration of an ADL episode	4 days	4 days	[8, 10–17]
Reduction in the frequency of ADL episodes by MDA	50 %	50 %	[33, 36, 37]
*Chronic Disease*			
Percentage in different clinical disease states	62.5 %	37.5 %	[7]
Percentage of chronic disease alleviated by MDA	10 %	15 %	[20, 30–35]

Due to the lack of region-specific data, a standard rate or proportion was utilized for each GPELF country. *ADL* acute adenolymphangitis, *MDA* mass drug administration

For LF, acute disease refers to recurring attacks of acute adenolymphangitis (ADL), defined by symptoms of pain, tenderness, local swelling, and warmth in the groin or limbs with constitutional symptoms (such as fever, nausea, and vomiting) [8–10]. It was assumed that these episodes last on average 4 days [8, 10–17]. Seventy percent of hydrocele and 95 % lymphedema patients were assumed to experience ADL episodes, with an average of two and four episodes per year respectively [8, 10–17]. These assumptions were varied in the sensitivity analysis.

### Calculating the health impact of the GPELF

#### Benefit Cohort Populations and the impact of treatment

**Fig. 3:**
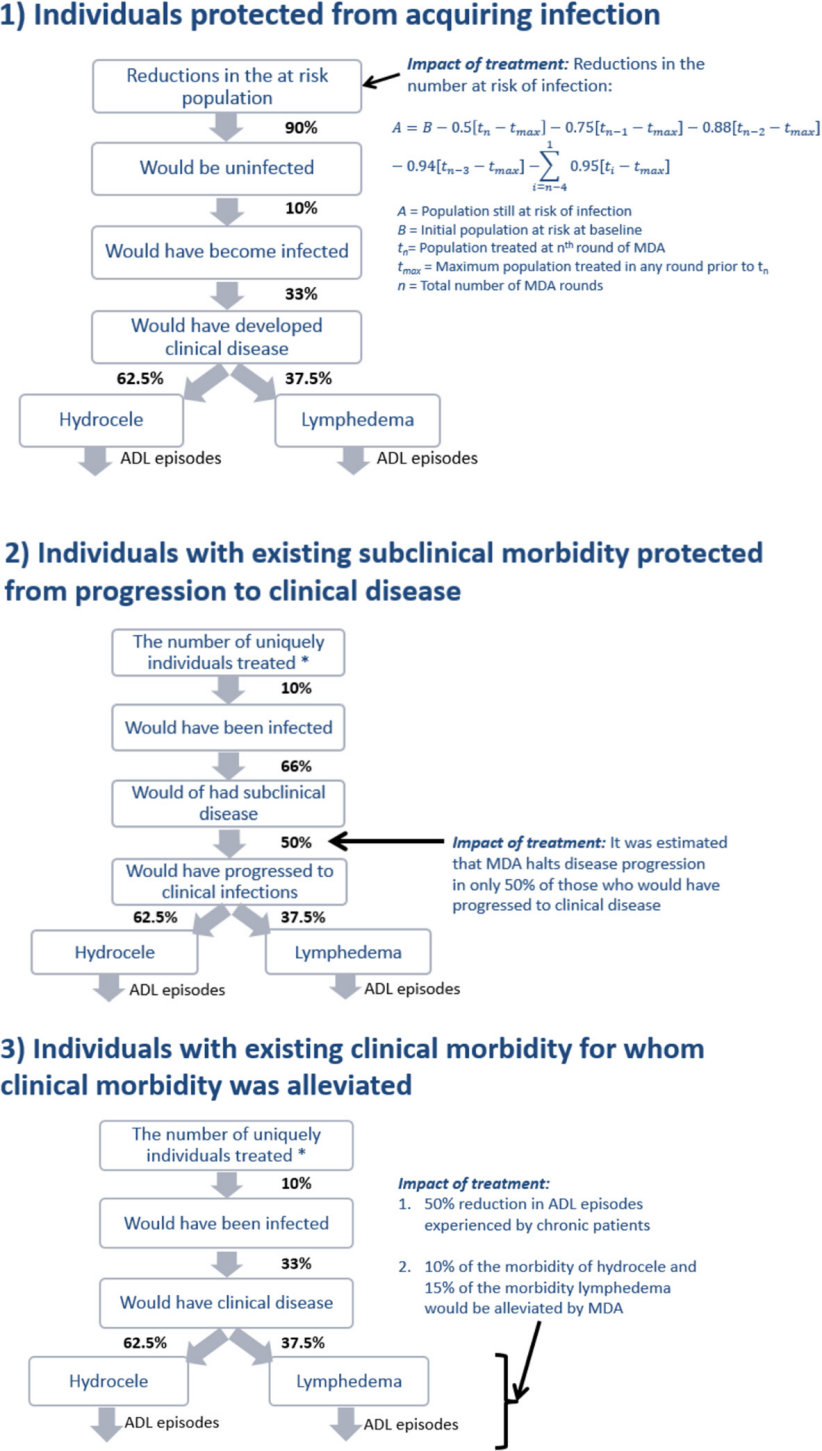
Schematic representation of the benefit cohorts and the assumed impact of treatment. *The number of uniquely treated individuals in any one country was assumed to be the maximum number of individuals treated in any single MDA for each country

For this analysis, the only individuals assumed to be incurring a health (and later, economic) burden due to LF were those with clinical disease. Consequently, three broad groups of individuals were recognized to have benefits from the MDA treatment provided under the GPELF (Fig. 3):

#### Benefit Cohort 1: individuals protected from acquiring infection

**Fig. 4:**
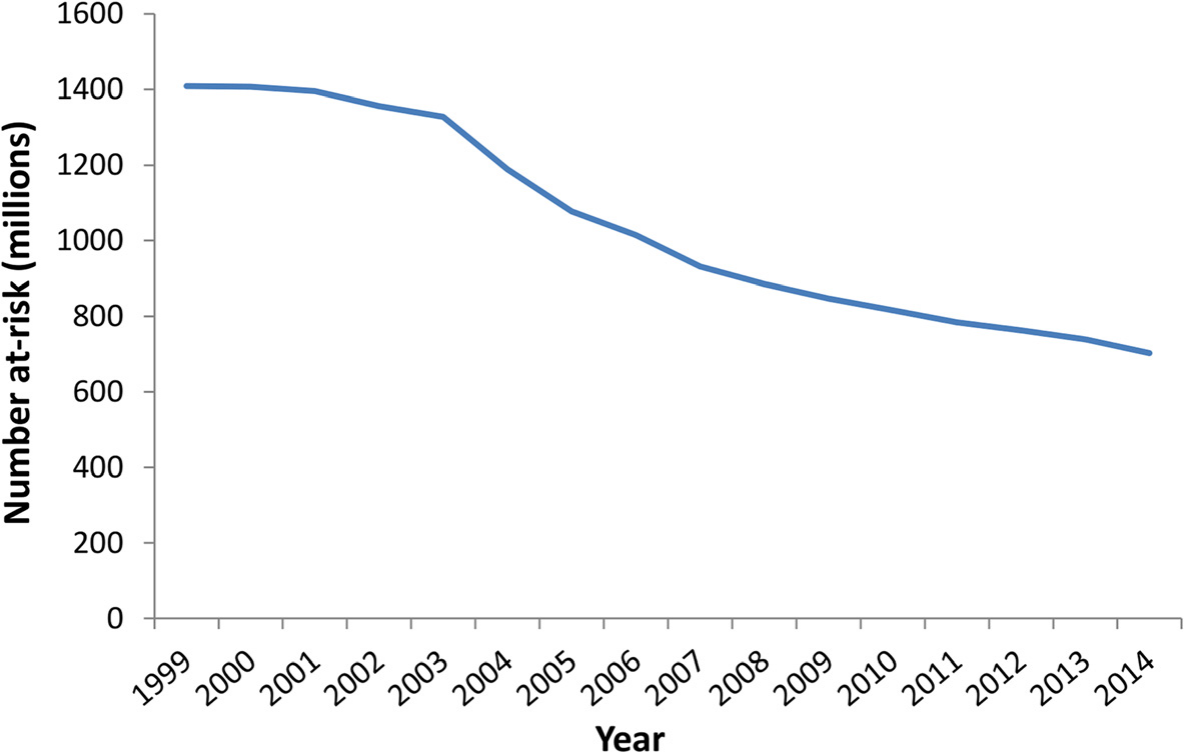
The estimated decline in the number of people at-risk of LF infection over time. The reductions were projected using the model presented in [18] and Fig. 3. Since a few countries are still doing mapping/have not started, the numbers at-risk remain incompletely defined. If a country has passed the Transmission Assessment Survey (TAS) in all of its implementation units it was assumed to have an at-risk population of zero (from that point forward)

Since the beginning of the programme in 2000 through the end of 2014, over 5.6 billion doses of anthelmintics have been administered to populations in 61 of the endemic countries (Table 4 and Fig. 1). This mass treatment will have had a major impact on the rate of transmission and consequently the number of people still at-risk of infection (and therefore the incidence of clinical infections) (Fig. 4).

**Table 4:** Percentage of clinical patients seeking treatment

Parameter	Hydrocele average estimate	Lymphedema average estimate	Source
Percentage of patients with ADL seeking treatment per episode	55 % (India: 70 %)	55 % (India: 75 %)	[8, 11, 14, 44, 46]
Percentage of chronic disease patients seeking treatment	20 % (India: 50 %)	30 % (India: 55 %)	[9, 44, 45]

Based on [4], though updated where appropriate. *ADL* acute adenolymphangitis

Though programmatic evidence suggests that effective transmission of LF might cease soon after the initiation of MDA, entomologic studies suggest that the decline in vector infection is more gradual [18]. We therefore approximated the reductions in the number at-risk for each country using a previously developed model which defines reductions in risk of infection among cohorts of treated populations following each treatment round (Fig. 3) [18]. As populations are treated, their risk of infection diminishes progressively after each MDA. The model was parametrised based on previous studies, which estimated a relationship describing an ‘average’ rate-of-decline of vector infection over five treatment rounds (pooling data from available studies [19–25]). This method uses the progressive decrease in vector infection rates as an indicator for decreased transmission, and, therefore, reduced population at risk of LF.

The model therefore accounts for a gradual decline in transmission, namely reductions of pre-control levels of 50, 75, 88, 94 and 95 % for the treated population following each of the first five MDA rounds respectively [18]. For this analysis, the reductions for the fifth MDA round onwards was set to 95 % and not the previous 100 % to account for potential residual transmission. These values were varied in the sensitivity analysis. The reductions in transmission are applied only to those treated and not the at-risk population as a whole (Fig. 3) [18].

The number of clinical infections prevented in this benefit cohort was estimated by assuming that in the absence of MDA, approximately 10 % of the projected population no longer at-risk would have become infected [2, 4], and 33 % of those would have developed clinical disease (Fig. 3). This is a modification of the previous analysis [4], which modelled newborns who are protected from infection over their lifetimes and other individuals protected from acquiring infection separately. The updated model [18] was used as it more accurately quantifies the impact of MDA on transmission and the reduction of the at-risk population as a whole.

The 17 countries that have passed the Transmission Assessment Survey (TAS) for all of their endemic districts/implementation units and stopped MDA were assumed to have an at-risk population of zero from that point onwards (before passing the TAS they had the transmission reduction rates described previously (see Fig. 3)).

#### Benefit Cohort 2: individuals with existing subclinical morbidity protected from progression to clinical disease

Based on previous studies it was assumed that approximately 66 % of individuals infected with LF have subclinical morbidity [1] and about 50 % of these would progress to overt clinical disease in their lifetimes [2, 4]. As in the previous analysis [2, 4], it was conservatively assumed that MDA halts disease progression in 50 % of those who would have progressed from subclinical to clinical disease [26].

Though the number of individuals treated in each MDA round for each country is known [5], it is not possible to estimate how many different individuals received treatment across multiple MDA rounds. Consequently, a conservative approach was taken and the number of uniquely treated individuals in any one country was assumed to be equal to the maximum number of individuals treated in any single MDA round [5].

#### Benefit Cohort 3: individuals with existing clinical morbidity for whom clinical morbidity was alleviated

There remains considerable uncertainty regarding the extent to which MDA improves the state of morbidity in those already suffering from hydrocele or lymphedema. However, several studies have provided preliminary evidence that repeated rounds of MDA may alleviate some LF clinical morbidity [20, 27–31]. As in the previous analysis [4], a conservative estimate of 10 % of the morbidity of hydrocele cases and 15 % of the morbidity of lymphedema cases being alleviated by MDA was assumed. However, due to the uncertainties surrounding these values, they were varied in the sensitivity analysis (ranging from 0 % alleviation up to 69–90 % based on the lower and upper boundaries cited by the literature [20, 30–35]).

Treatment is also known to reduce the frequency of ADL episodes experienced by chronic patients [33, 36, 37]. Due to the uncertainty surrounding this parameter, we assumed a conservative estimate of a 50 % reduction in the frequency of ADL episodes in chronic patients and varied this between 15 and 88 % in the sensitivity analysis [33, 36, 37] (Fig. 3).

### Calculating the total health and economic benefits of the GPELF

**Fig. 5:**
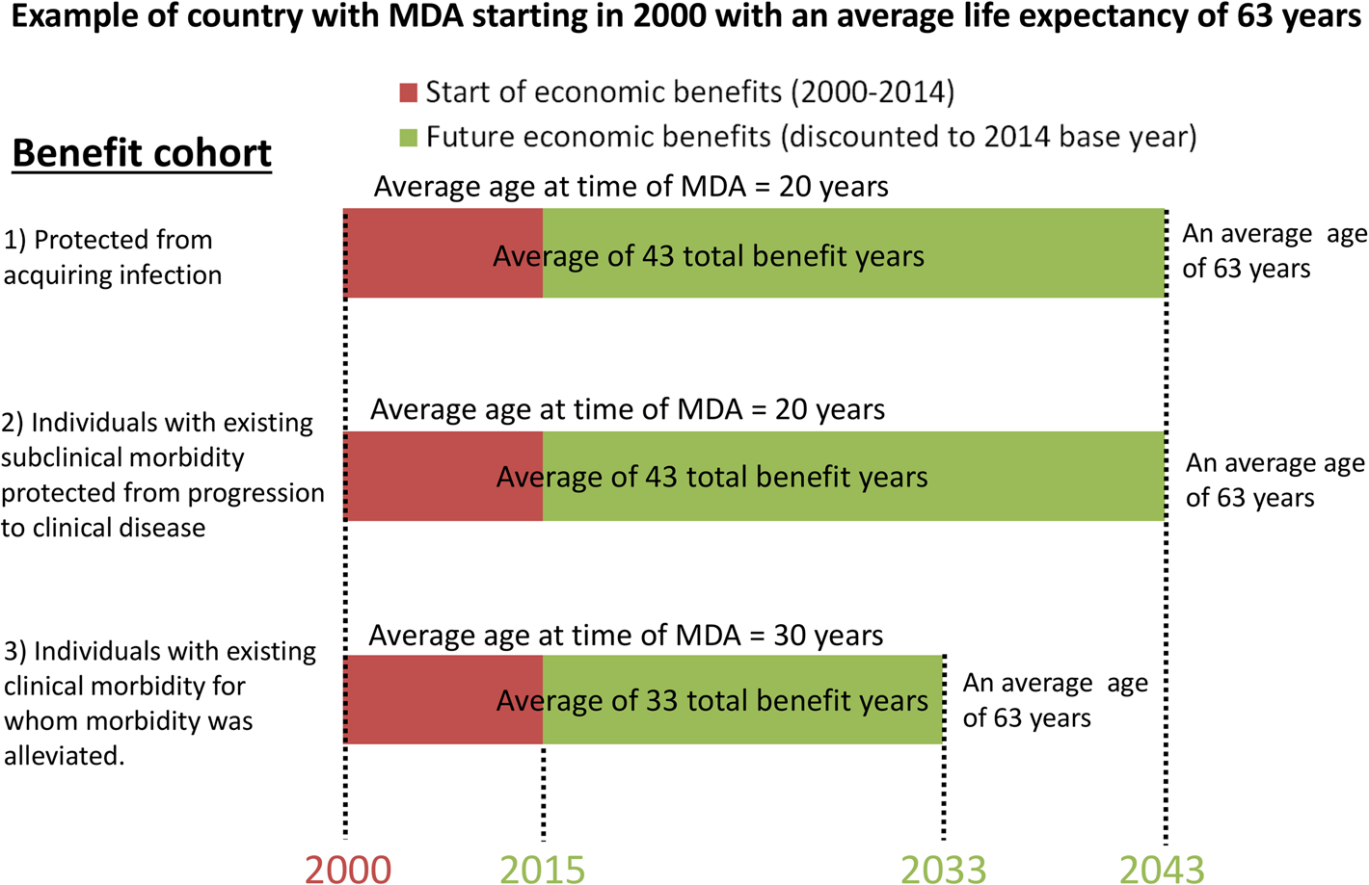
Duration of the health and economic benefits for the different benefit cohorts. The base year of the analysis was 2014. Health and economic benefits are calculated only for the benefit cohort populations receiving MDA between 2000 and 2014 (*red bar*); however, the benefits are gained until the end of their lifetime (*green bar*). For modelling purposes, single average ages were used to encompass the entire age range of individuals in each population benefit cohort accounting for the fact that, in reality, some individuals receiving treatment will be younger or older than the average age. The size of each benefit cohort decreases each year based on country and age-specific mortality rate. Figure based on [4]

In this paper we investigated the total health and economic impact of the GPELF for the three benefit cohorts defined in the previous section for the years 2000–2014. In the absence of transmission, protection from LF infection and disease progression is lifelong, thus it is necessary to aggregate the total health and economic benefit gained over the remaining lifetime of each benefit cohort population (Fig. 5). Life expectancy estimates were taken from existing tables [38]. The calculations were performed for each country individually to account for country-specific differences (such as life expectancy, mortality rates, medical expenses, wages etc.). All calculated economic benefits were discounted at 3 % to the base year of 2014.

The perspective of the analysis was that of the society as a whole – including the costs incurred by the patients (both direct and indirect costs), and national health systems.

#### Duration of health and economic benefits

The duration of the health and economic benefits was dependent on A) the age of onset of clinical disease (assumed to be 20 years old [2] in each country), B) the average life expectancy (differing by country), and C) the mean age at which an individual received MDA treatment (differing by cohort). For the latter the following assumptions were made (based on [4]):

- Individuals protected from acquiring infection (Benefit Cohort 1): The model assumes that the average age at treatment (and thus protection) for this cohort is 20 years.
- Individuals with subclinical morbidity at the time of MDA (Benefit Cohort 2): Though subclinical infection is common in early childhood, the model assumes that the average age at treatment (and thus protection) for subclinical patients is also 20 years.
- Individuals with clinical disease at the time of MDA (Benefit Cohort 3): The model assumes these individuals to be 30 years old on average when they receive MDA. This estimate implies that clinical disease patients have been living with their condition for an average of 10 years, since onset of clinical disease is assumed to be 20 years of age.

In the model, these average ages (20, 20 and 30 years) were used to encompass the entire age range of individuals within each benefit cohort population at the time of treatment – accounting for the fact that, in reality, some individuals receiving treatment will be younger or older than the average age (Fig. 5) [4].

#### Population size

This study projects the total health and economic impact of the first 15 years of the GPELF by aggregating the benefits over the lifetime of the 2000–2014 benefit cohorts. No projections are made for the expansion of MDA programmes after 2014 or their resulting benefits. The size of the benefit cohort populations were set to decrease dictated by the country-specific mortality rates [39]. Because the model population size decreases over time, the economic-benefit denominator was analysed in person-years (the sum of each year lived by each individual in each benefit cohort population).

### DALYs averted

The number of disability-adjusted life years (DALYs) averted due to the GPELF (2000–2014) was estimated by applying the 2010 Global Burden of Disease (GBD) disability weight of 0.11 (0.073–0.157) [40] to the number of clinical (symptomatic) LF person years averted (sum of each year lived by each individual in the benefit cohort population (Fig. 5)). No distinction in the disability weight was made between hydrocele and lymphedema. Based on the methodology employed by the 2010 GBD study we did not apply a discount rate or age weighting to the DALY estimates [41].

### Economic costs prevented

All of the economic benefits from MDA were assumed to arise from individuals within the benefit cohorts (i.e. individuals who would have had clinical disease without the GPELF). The economic costs prevented are comprised of both:

- Direct costs: representing the costs associated with medical resource utilization, which include the cost borne by the patients (i.e. for the medicines and transport etc.) and the health system (i.e. for health care personnel and capital resources etc.) [42].
- Indirect costs: defined as the expenses incurred from the cessation or reduction of work as a result of the morbidity and mortality associated with a given disease [42].

#### Direct costs

##### Patient medical expenses

Medical expenses are incurred by patients with clinical morbidity seeking treatment (Table 4). Patients were assumed either to go to a public or private health facility or to perform self-treatment/use traditional healers (Additional file 2: Table S1). Chronic disease sufferers who seek treatment following bouts of severe pain and swelling were assumed to receive anti-inflammatory medicines (ibuprofen (400 mg), and paracetamol (500 mg)). Patients seeking treatment for ADL were assumed to receive the same anti-inflammatory medicines and antibiotics (amoxicillin (500 mg)) [43]. We conservatively assumed that the standard treatment course was 8 days.

Both the proportion of clinical patients who seek treatment (Table 4) and the treatment source (Additional file 2: Table S1) were dependent on the morbidity type. In India, a higher proportion was assumed to seek treatment and to go to health facilities (Table 4). Due to lack of data, a lower global estimate was used for the other GPELF counties (Table 4). It was also assumed that the chronic hydrocele and lymphedema patients seeking treatment would do so an average two and three times per year respectively.

As in the previous analysis [4] it was assumed that for individuals seeking treatment at health facilities (both public and private), 50 % of their total medical expenses was for the medicines, 30 % for consultation fees and 20 % for transport, food, and accommodation [11, 44–46]. Median international reference prices for a course of amoxicillin (500 mg), ibuprofen (400 mg), and paracetamol (500 mg) were collected from the Management Sciences for Health International drug price indicator guide [47]. These were adjusted to obtain country-specific cost estimates for both public and private treatment using the Median Price Ratios from the relevant Health Action International database [48]. For countries not listed, the lowest value within the same region was used as a proxy. These medicine costs (which are 50 % of the total cost) were doubled to arrive at the estimated total medical expenses. For self-treating individuals, only the private medicine costs for ibuprofen and paracetamol were attributed to the total medical expenses. A summary of the medical expenses for each region is shown in Additional file 2: Table S2.

##### Health system costs

Reductions in the number of clinical cases will also reduce the amount of medical care that needs to be provided for LF, and consequently financial costs to the health systems of endemic countries. To estimate these patient-service savings, country-specific costs for a consultation at a rural primary health centre were gathered from the WHO CHOICE database [49]. These costs were then multiplied by the number of clinical LF cases averted, the percentage that seek treatment and go to a public health facility (Table 4 and Additional file 2: Table S1), and the average number of treatment visits per year (Table 3). Note that the WHO CHOICE database was updated in 2011, hence the health system costs are different from [4]. A summary of the average health system costs per visit for each region is shown in Additional file 2: Table S2.

Due to the absence of data regarding the cost and actual number of hydrocele surgeries performed in endemic countries, it was not possible to incorporate their costs into the analysis [43]. However, the proportion of total direct costs related to hydrocele surgeries would likely be small due to the still inadequate, relatively low frequency of hydrocelectomies.

#### Indirect costs

##### Lost wages

Clinical LF is debilitating, and patients are unable to work the same number of hours as equivalent workers not experiencing clinical symptoms (although chronic disease is less debilitating than acute (ADL) episodes). Reduced work hours and economic activity due to LF morbidity results in income loss for clinically infected individuals (an indirect cost).

Our assumptions regarding the reduction in productivity due to LF morbidity (Table 5) were based on a number of studies reporting the average number of hours worked by those with clinical disease compared to LF disease free controls (therefore accounting for employment rates in the local study setting). The difference between cases and controls is the percentage of working hours lost due to LF morbidity. It should be noted that these cost-of-illness studies must be interpreted with a degree of caution, as they are often highly sensitive to methodological assumptions; hence we employed conservative values (Table 5) and varied them over a wide range in the sensitivity analysis. We included both the productivity lost due to chronic disease and ADL episodes within our calculations of the indirect costs (Table 5).

**Table 5:** Economic model parameters

Disease type	Parameter	Hydrocele average estimate	Lymphedema average estimate	Sources
Acute	Average patient medical expenses per ADL episode	Country-specific (US$1.18^a^)	Country-specific (US$1.18^a^)	[8, 11, 44–48]
Chronic	Average patient medical expenses for chronic disease per year	Country-specific (US$0.70^ac^)	Country-specific (US$1.05^ac^)	[8, 11, 44–48]
Acute	Percentage of work hours lost per day during an ADL episode	75 %^b^	75 %^b^	[12, 14, 16, 87]
Chronic	Percentage of work hours lost due to chronic disease	15 %^b^	19 %^b^	[12, 43 , 45, 46]
Acute & chronic	Average wage per day (minimum of sources (Table 6))	Country-specific (US$1.50^a^)	Country-specific (US$1.50^a^)	[53–56]
Chronic	Work days per year	300^b,d^	300^b,d^	

Based on [4], though updated where appropriate

^a^Weighted average over all GPELF countries (based on the benefit cohort population size in each country) (Additional file 2: Table S2 and Table 6)

^b^Global estimate indicates a standard rate or proportion was utilized for each GPELF country. This is primarily due to a lack of supporting country-specific data

^c^Chronic hydrocele and lymphedema patients are assumed to seek treatment on average two and three times a year respectively

^d^Assume an average 6 day work week, 50 weeks of the year

Costs are expressed in US$ 2014 prices

*ADL* acute adenolymphangitis

As in [4], the indirect cost estimates were calculated using the human capital approach (i.e. based on the income foregone as a result of illness (any hour not worked is counted as an hour lost)) [50–52]. It should be acknowledged that approximating the income for individuals with LF is difficult; for example many of those infected are subsistence farmers who do not participate in the formal labour market [4]. For this reason, a combination of four wage sources was used to estimate the country-specific fair market value of time for an agricultural worker with LF infection (as in [4]): A) The International Labour Organization’s LABORSTA database (which lists country-specific average wages for agricultural field workers) [53]; B) The World Bank’s World Development Indicators (which lists a country-specific average value added per agricultural worker) [54]; C) The International Labour Organization’s Minimum Wages Database [55] and D) United States Department of State Country Reports on Human Rights Practices (which both list country-specific minimum wages) [56]. To ensure a conservative estimate, the lowest wage value was used for countries listed by more than one of the sources. For countries not listed by any of the four sources, the lowest value within the same region was used as a proxy. It was assumed that all individuals at-risk of or infected with LF would have been potentially economically active otherwise, and would work 300 days per year (six days a week, 50 weeks a year), 8 hours a day. A summary of the wages for each region and source is shown in Table 6.

**Table 6:** Summary of the different wage sources

WHO region	ILO LABORSTA (farm worker average wage)	World Bank (average value per agricultural worker)	ILO (minimum wage)	US State Department (minimum wage)	Overall average – maximum of sources^a^	Overall average – minimum of sources^a^
AMRO	$6.90	$27.36	$5.27	$5.17	$27.36	$5.11
AFRO	$1.92	$8.18	$2.06	$2.16	$9.05	$1.01
EMRO	$4.84	$18.26	$3.19	$5.00	$18.26	$3.00
WPRO	$5.36	$9.06	$6.81	$6.74	$10.70	$4.98
SEARO	$1.80	$3.47	$1.77	$3.75	$4.04	$1.47
Average	$2.02	$5.10	$2.10	$3.42	$5.84	$1.50

*AMRO* Region of the Americas, *AFRO* African Region, *EMRO* Eastern Mediterranean Region, *WPRO* Western Pacific Region, *SEARO* South-East Asia Region, *ILO* International labour organization

Results in this paper use the “Overall average – minimum of sources” estimates

Values shown are weighted averages (based on the benefit cohort population size in each country)

^a^The overall maximum and minimum averages were estimated from all sources for each country individually, and then averaged by region (which is why the values are smaller than the regional database averages)

Costs are expressed in US$ 2014 prices

For countries not listed in the database, the lowest value within the same region was used as a proxy

#### Cost standardization

To standardize the treatment prices and wages over different time periods, all estimates were adjusted for inflation using the gross domestic product price deflator and are expressed in US$ 2014 prices [57]. Estimates were then converted from local currencies to US dollars using the average exchange rates for 2014 [58].

#### Discounting

The base year for calculating economic benefits was set to 2014. The economic benefits were discounted at 3 % per year from this time point in accordance to guidelines set by WHO-CHOICE [59]. The discount rate was varied (0–6 %) within the sensitivity analysis [59]. No discount rate was applied for the years prior to the base year (2014). The DALYs averted/health benefits were not discounted in this analysis [41].

### Sensitivity analysis

Sensitivity analysis was performed in the following areas; A) Disease progression and incidence rates, B) Patient medical expenses and treatment seeking behaviour, C) Lost productivity and wages, D) The discount rate and E) Impact of treatment. The parameters and ranges investigated are shown in Table 7.

**Table 7:** Summary of the sensitivity analysis

Parameter	Hydrocele average estimate	Lymphedema average estimate	Sources
*Disease Progression & Incidence Rates*			
Percentage of clinical patients who experience ADL episodes per year	70 % (45–90 %)	95 % (90–95 %)	[8, 10–17]
Frequency of ADL episodes for clinical patients (in absence of MDA)	2 (0–7) per year	4 (0–7) per year	[8, 10–17]
Average duration of an ADL episode	4 (1–9) days	4 (1–9) days	[8, 10–17]
Disability weight for symptomatic LF infection	0.11 (0.073–0.157)	0.11 (0.073–0.157)	[40]
Mean age of the benefit cohorts (years)	Cohort 1: 20 (30)	Cohort 1: 20 (30)	
	Cohort 2: 20 (30)	Cohort 2: 20 (30)	
	Cohort 3: 30 (40)	Cohort 3: 30 (40)	
*Patient Medical Expenses and Treatment Seeking Behavior*			
Percentage of patients with ADL seeking treatment per episode	55 % (55–70 %)	55 % (55–70 %)	[8, 11, 14, 44, 46]
	India 70 % (70–98 %)	(India 75 % (75–98 %))	
Percentage of chronic disease patients seeking treatment	20 % (20–50 %)	30 % (30–55 %)	[9, 44, 45]
	India: 50 % (41–80 %)	India 55 % (48–100 %)	
Average patient medical expenses per ADL episode	+−20 % of baseline value	+−20 % of baseline value	[8, 11, 44–48]
Average patient medical expenses for chronic disease per year	+−20 % of baseline value	+−20 % of baseline value	[8, 11, 44–48]
*Lost Productivity & Wages*			
Work days per year	300 (261–365) days	300 (261–365) days	
Percentage of work hours lost per day during an ADL episode	75 % (50–93 %)	75 % (50–93 %)	[12, 14, 16, 87]
Percentage of work hours lost due to chronic disease	15 % (9–24 %)	19 % (11–31 %)	[12, 43, 45, 46]
*Discounting*			
Discount rate	3 % (0–6 %)	3 % (0–6 %)	[59]
*Impact of Treatment*			
The reduction in transmission experienced by the treated population	Year 1: 50 % (35 %)	Year 1: 50 % (35 %)	[18]
	Year 2: 75 % (53 %)	Year 2: 75 % (53 %)	
	Year 3: 88 % (62 %)	Year 3: 88 % (62 %)	
	Year 4: 94 % (66 %)	Year 4: 94 % (66 %)	
	Year 5 95 % (67 %)	Year 5 95 % (67 %)	
Reduction in the frequency of ADL episodes by MDA	50 % (15–88 %)	50 % (15–88 %)	[33, 36, 37].
Percentage of chronic disease alleviated by MDA	10 % (0–90 %)	15 % (0–69 %)	[20, 30–35]

Based on [4], though updated where appropriate. *ADL* acute adenolymphangitis, *MDA* mass drug administration

### Ethical approval

This study does not require ethical approval.

## Results

Between 2000 and 2014, the GPELF has delivered 5.6 billion treatments to over 763 million people at least once, in 61 of the 73 identified endemic countries.

Using the model presented in Fig. 3 and the corresponding treatment data, we projected that the total benefit cohort of the GPELF (2000–2014) would consist of 46 million individuals (Table 8) (i.e. these individuals would have had clinical disease without the GPELF interventions). Of these, 21 million (45 %) were in Benefit Cohort 1 (would have acquired LF and subsequently progressed to clinical disease but were protected from infection due to reductions in transmission by MDA). The remaining 25 million were individuals who were already infected at the time of MDA treatment but benefited from halted disease progression (Benefit Cohort 2: 12.5 million (27 %)), or alleviated clinical disease (Benefit Cohort 3: 12.8 million (28 %)) (Table 8).

**Table 8:** Summary of the benefit cohorts

Benefit cohort	Population size (*millions*)	Average age at MDA treatment	Average years of health and economic benefit^a^	Person-years (*millions*)^b^
1. Protected from acquiring infection	21	20	45.6	938
2. Subclinical morbidity prevented from progressing	12.5	20	45.6	551
3. Alleviated clinical disease	12.8	30	35.6	450^c^
Total	46	**-**	**-**	1,939

^a^Based on a global weighted average life expectancy of 64.6 years (weighted based on the benefit cohort population size in each endemic country)

^b^The sum of the number of years lived by each individual in the benefit cohort population. (Equal to the benefit cohort population multiplied by the average years of health and economic benefit (after adjusting for mortality))

^c^Includes both those with alleviated chronic disease and these with reduced frequency of ADL episodes (Fig. 3)

### Health benefits

Administering MDA to such a large population has produced substantial health benefits over the first 15 years of the GPELF. We estimate that the programme would prevent 1,592 million years lived with chronic disease (36 million chronic cases) and 4,689 million ADL episodes over the lifetimes of the benefit cohorts (Table 9). This translates into approximately 175 (116–250) million DALYs averted – range depending on the 95 % uncertainty range of the disability weight (Table 9).

**Table 9:** Projected health impact of the GPELF (2000–2014) over lifetime of the benefit cohorts

Benefit cohort	Number of chronic cases averted (*millions*)	Years of chronic disease averted (*millions*)	Number of acute (ADL) episodes averted (*millions*)	DALYs averted (*millions*)^a^
1. Protected from acquiring infection	21	938	2,157	103 (68–147)
2. Subclinical morbidity prevented from progressing	12	551	1,267	62 (41–88)
3. Alleviated clinical disease	3	104	1,264	11 (8–16)
Total	36	1,592	4,689	175 (116–250)

^a^Range based on the 95 % uncertainty interval of the disability weight (Table 7)

See Additional file 2: Table S3 for the results stratified by WHO region. *ADL* acute adenolymphangitis

The total health impact was smallest for the *Alleviated clinical disease* cohort (Benefit Cohort 3) despite its comparable population size (Table 8). This is due to the fact that the majority of this cohort were only experiencing reductions in the frequency of ADL episodes and not fully alleviated chronic disease (Table 9) (due to the assumed effects of treatment on morbidity (Table 3 and Fig. 3)). Furthermore, as members of this cohort were assumed to be older at the time of MDA, they have fewer years of economic benefit (and consequently a lower number of person-years in the benefit cohort (Fig. 5 and Table 8)).

### Economic benefits

We estimated that on average, each individual in the benefit cohort will potentially avoid losing US$2 095 over his/her lifetime (US$50 per year) (Tables 10 and 11). This is equivalent to the income earned by working 33.3 days per year (Table 10). Though these sums and averages vary between different regions (Table 10 and Additional file 2: Table S4), much of the variation can be attributed to higher wages in non-AFRO/SEARO countries (Table 6) (where only 4 % of the treatments during this 15-year period have been given (Fig. 1)). It is important to note that each year after 2014, the economic benefit is discounted by 3 % per year.

**Table 10:** Costs prevented per individual of the benefit cohort population

WHO region	Annual medical expenses averted per person within the benefit cohort^a^	Annual wage loss prevented per person within the benefit cohort^a^	Annual economic benefit per person within the benefit cohort^a^	Equivalent number of days income per year^ab^
AMRO	$1.75	$158.49	$160.24	31
AFRO	$0.77	$30.15	$30.92	31
EMRO	$0.62	$102.54	$103.17	34
WPRO	$1.32	$160.07	$161.39	32
SEARO	$1.91	$50.37	$52.27	36
Average	**$1.52**	$48.48	$50.00	33.3

*AMRO* Region of the Americas, *AFRO* African Region, *EMRO* Eastern Mediterranean Region, *WPRO* Western Pacific Region, *SEARO* South-East Asia Region. Costs are expressed in US$ 2014 prices

^a^Does not include reduction in costs to the health system (Additional file 2: Table S4)

^b^Annual economic benefit per person within the benefit cohort divided by the average wage estimate for that region (Table 6)

**Table 11:** Total costs prevented for individuals and the health systems over lifetime of the benefit cohorts

Benefit cohort	Direct costs for individuals prevented - medical expenses (*millions*)	Indirect costs for individuals prevented - lost wages (*millions*)	Direct costs for the health system prevented (*millions*)	Total costs prevented (*billions*)	Lifetime benefit per individual within the benefit cohort^a^
1. Protected from acquiring infection	$1,376	$52,513	$1,813	$55.70	$2,569
2. Subclinical morbidity prevented from progressing	$818	$31,273	$1,063	$33.2	$2,572
3. Alleviated clinical disease	$744	$10,210	$664	$11.6	$855
Total	$2,938	$93,996	$3,540	$100.5	$2,095^b^

^a^Does not include the economic benefit to the health system

^b^Weighted average

See Additional file 2: Table S4 for the results stratified by WHO region. Costs are expressed in US$ 2014 prices

In total, we estimated that due to the notable health impact of the GPELF, individuals in the benefit cohort would avoid losing US$96.9 billion in costs over their lifetimes. This total amount results from summing the prevented patient medical expenses (US$2.9 billion) and potential income loss (US$94 billion) prevented for these individuals (Table 11). Reductions in the number of clinical LF cases will also reduce the patient-service costs for the public health system. We estimated that approximately US$3.5 billion in health system costs will be saved over the lifetime of the benefit cohort populations. Consequently, the total economic impact for both the individuals in the benefit cohort and the health system was estimated to be potentially US$100.5 billion (Table 11).

#### Economic benefits by cost type

**Fig. 6:**
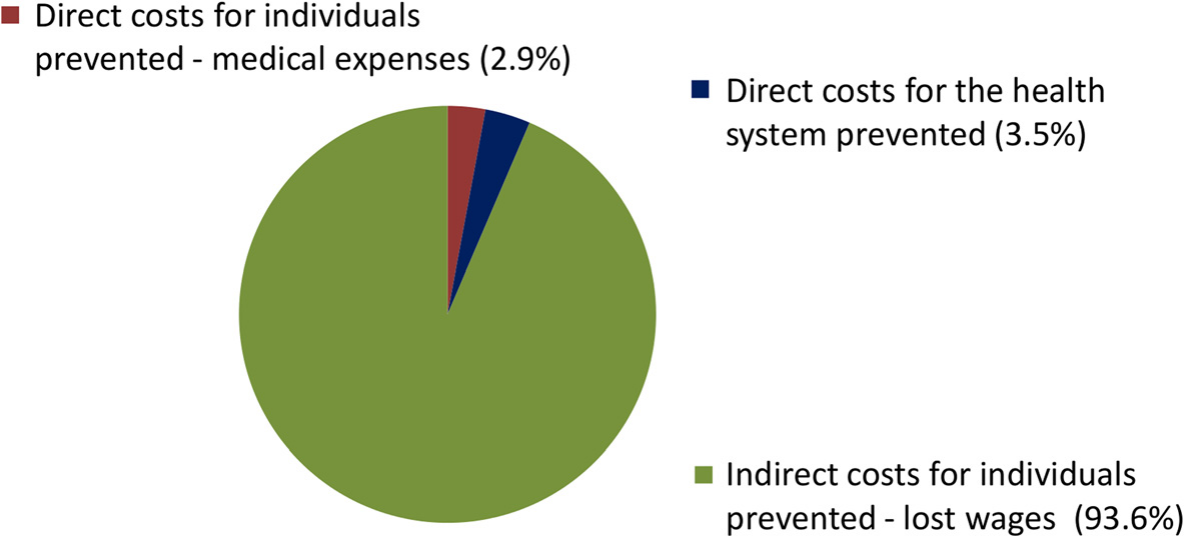
Total economic benefit disaggregated cost type

Approximately 93.6 % of the projected total economic benefit can be attributed to the prevention of reduced productivity and subsequent income loss – an indirect cost (Fig. 6 and Table 11). The remaining benefit was related to prevented patient medical expenses (2.9 %) and health system costs prevented (3.5 %) – both direct costs. The relatively low proportion for prevented patient medical expenses was attributable to the low frequency of treatment seeking behaviour and relatively inexpensive medicine packages relative to the day-to-day accumulation of lost income from reduced economic activity.

#### Economic benefits by morbidity type

**Fig. 7:**
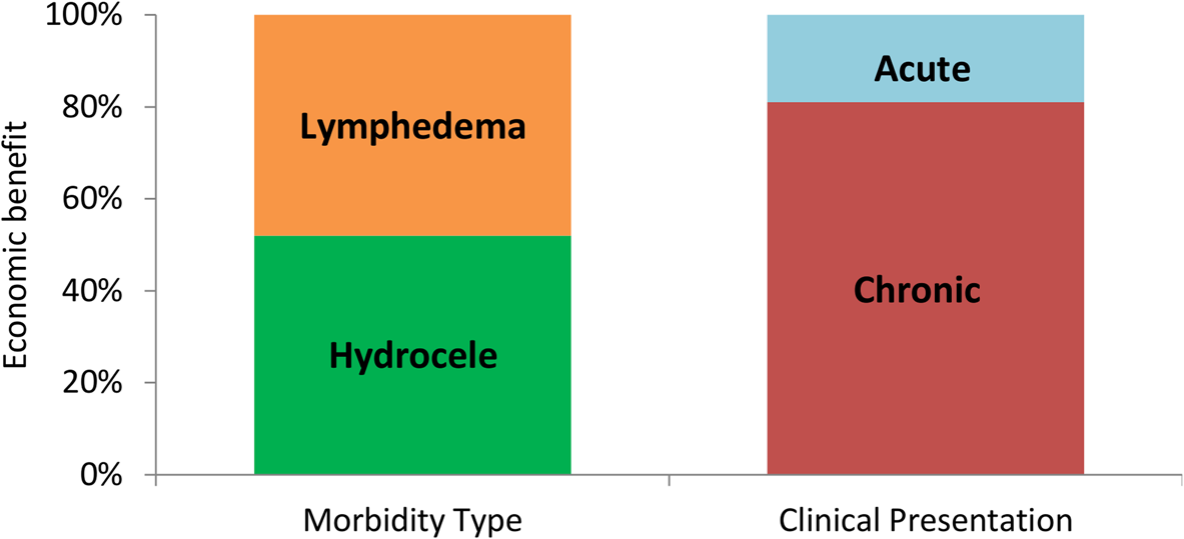
Total Economic benefits by morbidity type, and clinical presentation

The total economic benefits accrued by populations protected from hydrocele were approximately equal to those from lymphedema (Fig. 7). This result occurred because the greater average disability of lymphedema patients (Table 3) offsets the estimated higher proportion of clinical disease patients with hydrocele (62.5 % vs 37.5 %).

Prevented chronic disease accounted for about 81 % of the total economic benefits (Fig. 7). This is to be expected, as though acute episodes are more debilitating, they have a short duration (8–16 days per year) – whereas the chronic condition is a life-long disability (Table 3).

### Sensitivity analysis

To determine how sensitive the results were to variation in the input parameters and assumptions, the model projections were subjected to univariate sensitivity analysis (see Table 7 for a full description).

The projected impact on health was found to be very robust, with the number of DALYs averted only being reduced by 17 % when assuming more conservative reductions in transmission, and 18 % when increasing the mean age of the benefit cohorts by 10 years (Additional file 2: Table S5). When assuming that MDA has no impact regarding alleviating established chronic disease, the number of DALYs averted only decreased by 7 % (and increased by 37 % when using the upper range of reported values for this parameter) (Additional file 2: Table S5). The number of DALYs averted was sensitive to the assumed disability weight. However, even when using the lower bound (0.073) for the disability weight [40], we still projected that 116 million DALYs would be averted.

**Fig. 8:**
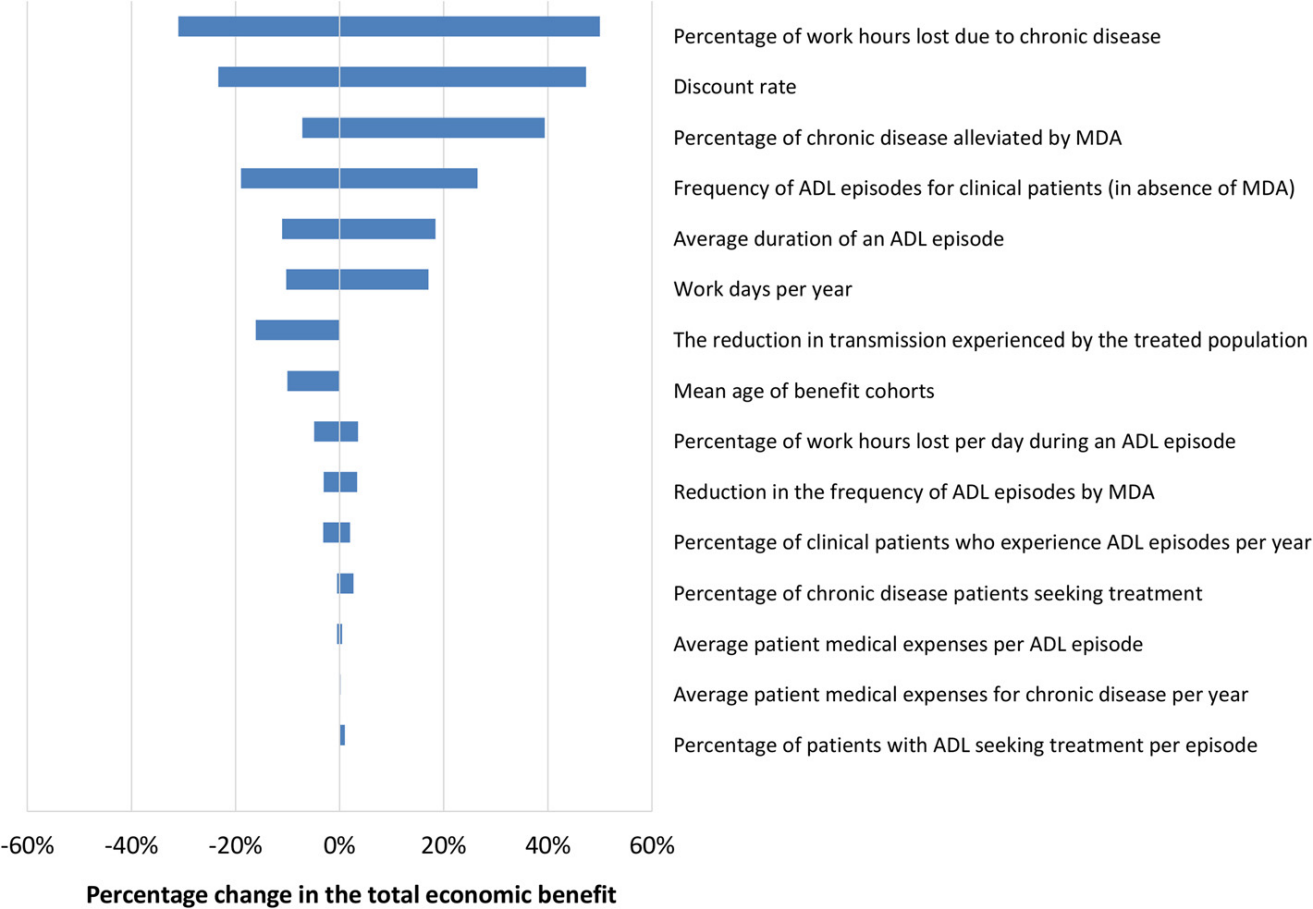
Tornado plot illustrating the impact of the sensitivity analysis on the estimated total economic benefit of the GPELF (2000–2014). The parameter ranges investigated are shown in Table 7. Results stratified by cost type are shown in Additional file 2: Table S6

The sensitivity of the economic benefit of the GPELF to the different parameters is shown in the tornado diagram in Fig. 8 (which illustrates the percentage change in the total economic benefit when changing each of the model parameters in Table 7). The results were most sensitive to the assumed percentage of work hours lost due to chronic disease and when using the lower bound the total economic benefit decreased by 31 % (Fig. 8). The Figure also illustrates that if a lower discount rate was assumed that the economic benefit would be even larger (and vice versa). The sensitivity analysis stratified by cost type is shown in Additional file 2: Table S6.

## Discussion

These results further highlight that LF is responsible for not only a severe physical burden but also a considerable economic burden (from both medical expenses and loss of income-generating activity). The notable and sustained losses in labour productivity for clinical LF patients make it even harder for those living in endemic areas to escape from poverty without the aid of MDA interventions.

We projected that due to the first 15 years of the GPELF 36 million chronic cases would be averted (Table 9). Over the lifetime of the benefit cohort, this corresponds to 175 (116–250) million DALYs being averted (Table 9).

We estimated that, as a result of the first 15 years of the GPELF, 46 million individuals will gain economic benefits due to prevented clinical disease (Table 8) and that members of this benefit cohort would avoid losing US$96.9 billion in costs (an average of US$2 095 per person) over their lifetime due to the GPELF (Table 11). For individuals within the benefit cohort this is equivalent to the income earned from working 33.3 days (or 11 % of their average annual income (Table 10)). In addition to the economic benefits to individuals, we projected US$3.5 billion in health system costs will be saved in endemic areas (Table 11). Such savings are critical for health systems in resource poor settings. Furthermore, it is important to acknowledge that the analysis did not account for the decreasing need for hydrocele surgeries due to the MDA, and is therefore underestimating the economic benefits to health systems. The total economic impact for both the individuals and the health systems was estimated to be US$100.5 billion.

### Comparison to past estimates

A previous analysis by Chu et al. [4] estimated that following the first 8 years of the GPELF (2000–2007), US$21.8 billion of economic benefits will be gained in MDA-treated areas, in addition to US$2.2 billion in health systems savings. The difference in values between the studies are primarily due to the following reasons.

1. The analysis presented in this paper estimated the impact of the GPELF between 2000 and 2014. Since 2007, the programme has expanded to an additional 13 countries and delivered over 3.68 billion more treatments (Table 2 and Fig. 1) – therefore the economic impact has increased.
2. The number of individuals protected from acquiring infection (Benefit Cohort 1) was estimated using a model [18] defining reductions in the risk of infection among cohorts of treated populations (Fig. 3). This is a modification of the previous analysis [4] – as the model [18] was not developed at that time. Consequently, the size of the benefit cohort is substantially larger in this more recent analysis.
3. The assumed wages were higher than the values used in [4] (due to inflation and the fact that the databases used to obtain wage estimates are now more complete).
4. WHO Choice updated its cost database in 2011 and consequently, the assumed health care costs (per visit) are different from those in Chu et al. [4].

Other studies corroborate the finding that the largest economic burden of LF results from indirect costs (see [43, 60]). For example, it was estimated that in India, 8 % of the potential male labour input was being lost due to hydrocele and lymphedema [45]. This was subsequently valued at US$704 million per year [50]. In Ghana a similar values was reported, with more than 7 % of potential labour lost [9].

That we know of, there are very few other analyses of health and economic benefits of global health programs as a whole. This makes it difficult to compare these estimates to those of other global disease control programmes. However, it should be noted that large health gains can also be expected for programmes targeting other NTDs as we move towards the goals set be the London Declaration and WHO 2020 NTD roadmap [61, 62].

Within [61] it was estimated that if the London Declaration goals were met between 2010 and 2030, 46.4 new clinical LF cases would be averted. This appears comparable with our estimate of 33 million new cases being averted over the lifetime of benefit cohorts 1 and 2 (for the treatments given between 2000 and 2014) – though it is difficult to directly compare the estimates as they are analysing different time horizons.

### Reduced productivity and lost wages

The majority (93.6 %) of the economic benefit was projected to arise from potential income loss prevented (indirect costs). A variety of methods in valuing working time have been incorporated in economic analyses of similar tropical diseases, including the examination of minimum wages [63], average value added per agricultural worker [64], and proxies from prior studies in similar settings [65]. We used a combination of four wage sources to estimate a fair market value of time for an agricultural worker with LF infection (to ensure a conservative estimate, the lowest wage value was used for countries listed by more than one of the sources). For women, economic activity includes time spent on non-agricultural household chores (an opportunity cost). Our calculations used the human capital approach (which counts any hour not worked by a patient as an hour lost (Table 1)) [50–52].

However, approximating the value of the income loss is difficult for a variety of reasons [4, 52]. Firstly, the majority of this population is comprised of subsistence farmers who do not participate in the formal labour market, making it harder to place a value on their time [4]. Furthermore, although chronic patients may develop coping strategies to adapt to their condition and regain a degree of economic activity, many do so at the expense of lower-earning jobs that require less physical activity [9, 66] – which is difficult to quantify. In addition, many with severe morbidity will be confined to the home and likely have to give up income-generating activity completely [4]. It is also important to consider that many patients may be employed in occupations with higher wages than subsistence farmers (such as. weaving, mining, fishing) [4] and will therefore suffer a higher opportunity cost than assumed in this analysis; average incomes are also generally higher in urban areas, where up to a third of the LF burden in India exists [50, 67]. More socioeconomic research is necessary to yield greater accuracy in the estimates of the opportunity cost of LF and indirect economic benefits of the GPELF [4].

It should be noted that there was a substantial degree of variation in the wage estimates between the different databases (Table 6) and employment rates may vary across the different countries. To be conservative we used the lowest wage estimate for each country (Table 6). Furthermore, our analysis calculates indirect costs based on the equivalent hours and resulting wages lost from economic activity. However, this ignores the fact that as well as working less, LF patients may be less productive while at work [4]. For example, Ramu et al. found that though the reported time difference (hours worked) between LF infected and uninfected weavers was 15–20 %, the productivity gap was higher at 27 % [68]. Additional research on the actual productivity burden of LF, will be invaluable in developing more precise economic benefit estimates in the future [4]. The sensitivity of the results to the assumed percentage of work loss is shown in Fig. 8 and Additional file 2: Table S6.

### Additional benefits of the GPELF

#### Quality-of-life benefits

The primary health outcome of the analysis was the number of DALYs averted. However, it is important to note that the prevention of LF infection and clinical disease has led to additional quality of life benefits, which are not captured by the DALY disability weight for LF or the estimated lost wages. For example, the stigma associated with infection can prevent patients from playing a full role in society, often resulting in reduced marital prospects. This can result in adverse social and economic repercussions not only for the patient but also their family [69]. Furthermore, children may have to miss school in order to care for a family member with LF, and infected children will be more likely to miss school or drop out completely [70]. In addition, both those suffering from clinical disease and their caregivers are more likely to suffer from depression which is not currently quantified in the GBD estimates for LF – which has been shown to underestimate LF disease burden [69]. It should also be noted that DALYs fail to acknowledge the implications of context on the burden of disease, such as those for the poor –which is particularly important for NTDs [71]. Consequently, the true value of the GPELF will likely be higher than what is presented in this analysis.

#### Economic and health impacts on other co-endemic diseases

The GPELF uses highly effective, broad-spectrum anti-parasitic drugs (albendazole and ivermectin). Consequently, the programme has important ancillary benefits on other parasitic diseases (described in more detail in [2]):

- *Benefit for children and for women-of-child-bearing-age with intestinal parasites*: Albendazole and ivermectin are also used to control the soil-transmitted helminths (STH). Consequently, the GPELF is having a notable impact on these diseases (Table 12). It is important to note that LF programmes co-administrating albendazole and ivermectin will have a much higher impact on Trichuris [72] than STH control programmes using standalone-treatments [73, 74]. Furthermore, these community-wide programmes will have a higher impact on hookworm (for which the majority of worms are harboured by adults) [73, 75, 76].
- *Benefit for people with scabies*. Ivermectin is effective treatment for scabies and can cause the community prevalence to fall dramatically after a few rounds of treatment [77]. Cured individuals show improvements in sleep patterns and overall wellbeing and decreased incidence of skin infections and renal disease [78].
- *Benefit for co-endemic onchocerciasis areas*. Because of its broad geographic range, the GPELF has brought ivermectin treatment to millions of people living in onchocerciasis-endemic areas not previously targeted by onchocerciasis control programs (as these programmes generally focused only on communities where the prevalence of onchocerciasis exceeds 40 %) [79]. The GPELF is therefore likely contributing significantly to the elimination of onchocerciasis transmission.

**Table 12:** Potential impact on soil-transmitted helminths

Individuals reached	Target	Benefits
212 million children -minimal estimate-	Soil-transmitted helminths (intestinal parasites: hookworm, roundworm, whipworm)	Weight/height gain, learning ability, cognitive testing, school attendance, fitness, activity [88–91]
Assumptions and reasoning		
A) 1.1 billion treatments of albendazole given to children (aged 2–15 years old in countries treated with DEC and albendazole; 5–15 years old in countries using ivermectin and albendazole) in 61 countries during MDAs 2000–2014 [5, 6].		
B) The maximum number of children treated in any single MDA was determined for each country. The sum of these numbers indicates the minimum total number of children treated (212 million) [5, 6].		
Individuals reached	Target	Benefits
177 million women of childbearing age, not pregnant (minimal estimate)	Soil-transmitted helminths (intestinal parasites: hookworm, roundworm, whipworm)	Decreased anaemia [92], maternal mortality, infant mortality; increased infant birth-weight [93]
Assumptions and reasoning		
A) 947 million treatments of albendazole given to non-pregnant women-of-childbearing-age (aged 15–49 years old) in 61 countries during MDAs 2000–2014 [5, 6, 38].		
B) The maximum number of such women treated in any single MDA was determined for each country [5, 6]. The sum of these numbers indicates the minimum total number of women-of-childbearing-age treated (177 million). C) Since pregnancy is an exclusion criterion for LF treatment, the annual estimates thus derived were discounted by subtracting the estimated percent of the female population that is pregnant at any given time.		

Because individual country estimates of the prevalence and distribution of soil-transmitted helminths are generally not available, it was not possible to estimate directly the number of soil-transmitted helminths infections. However, a sizeable proportion of the albendazole and ivermectin treatments delivered for LF will have had a beneficial impact for children and women of childbearing age who harbour soil-transmitted helminths. The assumptions are outlined in [2]

### Limitations

#### Model assumptions and parameters

Though the number of individuals treated in each MDA round for each country is known [5], it is not possible to estimate how many unique individuals received treatment across multiple MDA rounds. Consequently, a conservative approach was taken and the number of uniquely treated individuals in any one country was assumed to be the maximum number of individuals treated in any single MDA for each country – which in most cases will be an underestimate.

The two different drug regimens used in the GPELF were assumed to be equally effective in their effects both on LF disease and on the filarial infections themselves [80].

For simplicity, a single average age was used to encompass the entire age range of individuals within each benefit cohort population at the time of treatment (with the recognition that some of those receiving treatment will be younger or older than the average age). However, realistically the average age would vary in different countries. Though this assumption will affect the economic benefit in any given year it is unlikely to affect the total benefit across the benefit cohort population’s lifetime (in the sensitivity analysis we found that if the mean ages were increased by 10 years, it would only decrease the total economic benefit by 10 %). It should be noted that some of the clinical cases would be averted further into the future then our model is projecting. Consequently, some of the economic benefits may be under-discounted.

The limited amount of country-specific primary data available somewhat limits the breadth of analysis presented in this paper though, much of the literature originates from India and sub-Saharan African countries where the majority of the benefit cohort population resides. Furthermore, due to a lack of regional data, many of LF disease-specific parameters (e.g. ADL frequency and duration, work hours lost) were attributed a global standardized estimate. However, the sensitivity analysis included a range of different values and the overall results appear robust.

Due to the way that the DALYs are calculated (with the same disability weight applied to any symptomatic infection), individuals in Benefit Cohort 3 (Fig. 3) with reduced ADL frequency but persistent chronic disease still had the same DALY weight applied to them. Furthermore, due to the absence of data no excess mortality of clinical patients was assumed. These assumptions may lead to an underestimation of the health impact of the GPELF (in terms of the number of DALYs averted).

#### Calculating the reductions in the at-risk population

The model [18] used to estimate the reductions in the at-risk population is dependent on data from WHO PCT Databank [5] regarding the numbers of people treated each year for LF. However, this data is self-reported by national programs, and while in many cases it has been found to be similar to independent coverage surveys, there are areas where frequent over-reporting has been identified (discussed further in [80]). Such over-reported coverage would lead the model to overestimate the number of infections averted by the GPELF. In addition, though data from all three mosquito genera transmitting LF were used to generate the model parameters, the biological differences of the vectors (or vector density) are not currently differentiated [18]. Furthermore, the model does not take into account the possibility of re-infection or resurgence of suppressed infections in areas that miss rounds of treatment or in areas where the coverage has decreased from the previous year [18]. On the other hand, such resurgence is uncommon [1], and many features of the model may be underestimating the effects of the GPELF on decreasing the number of at-risk individuals. For example, within the model, the reductions in transmission are applied only to those that have been treated. Consequently, it does not capture the indirect or herd effects of MDA to the populations covered as a whole [18]. The model is also based on the maximum number treated each round (which will underestimate the number uniquely treated). Furthermore, the model does not ‘zero out’ an at-risk population in the country until all of its implementation units have programmatically passed the TAS [18].

Though the model has important limitations, ultimately we are projecting that after the distribution of 5.6 billion treatments, the global number at-risk for LF infection has halved –which intuitively seems conservative (Fig. 4). The model was parameterized based on data derived from a broad range of entomological studies undertaken during active LF MDA programs in different geographical settings, providing a reliable estimate of changes in transmission potential under MDA pressure. A dynamic transmission model (such as [81–83]) would be more ideal and accurate for quantifying the impact of MDA on transmission (and would account for the indirect or herd effects of the GPELF) [84]. However, in order to be accurate this would likely require more detailed coverage data (at least at the implementation unit level), as opposed to the reported national coverage data available.

## Conclusion

Despite the limitations of any such analysis, this study indicates that substantial health and economic benefits have resulted from the first 15 years of the GPELF. We projected that due to the first 15 years of the GPELF 36 million chronic cases and 175 (116–250) million DALYs would be averted over the lifetime of the benefit cohort. It was estimated that due to this notable health impact, US$100.5 billion will potentially be saved over the lifetimes of those that would have had clinical disease without the GPELF. This total amount results from summing the patient medical expenses (US$3 billion), potential income loss (US$94 billion), and costs to the health system (US$3.5 billion) that were projected to be prevented over the lifetime of the benefit cohort. The results were subjected to sensitivity analysis and were most sensitive to the assumed percentage of work hours lost due to chronic disease (changing the total economic benefit between US$69.30–150.7 billion). In addition, the GPELF would have both further quality-of-life benefits and benefits on other co-endemic diseases (such as STH) as well – making the total health and economic value even greater than that presented here.

It is important to note that this large health and economic impact would be diminished if the control programmes were not continued until elimination is achieved and the infection was allowed to resurge.

Though this analysis made a number of assumptions, we attempted to be conservative in our approach. These results further highlight the value and importance of continued investment in the GPELF as additional resources will be necessary to assist the remaining countries in implementing programs for LF elimination [4, 85].

## Additional files

Additional file 1: Multilingual abstract in the five official working languages of the United Nations. (PDF 323 kb) Additional file 2: Table S1. The breakdown of where individuals receive treatment. **Table S2**. Regional specific patient medical expenses and health system costs. **Table S3**. DALYs averted stratified by WHO region. **Table S4**. Economic benefits for individuals and the health systems stratified by the WHO regions. **Table S5.** Impact of the sensitivity analysis on the projected health benefits of the GPELF (2000–2014). **Table S6**. Impact of the sensitivity analysis on the projected economic benefits of the GPELF (2000–2014). (DOCX 28 kb)
