# Supplementary material for: The health and economic benefits of the global programme to eliminate lymphatic filariasis (2000–2014)
Source: Infect Dis Poverty. 2016 May 24;5:54. doi: 10.1186/s40249-016-0147-4 (PMC4937583; doi:10.1186/s40249-016-0147-4)
Supplement: Additional file 1: — Multilingual abstract in the five official working languages of the United Nations. (PDF 323 kb) [file 40249_2016_147_MOESM1_ESM.pdf]

Translation of the abstract into the five official working languages of the United Nations

الفوائد الصحية والاقتصادية للبرنامج العالمي للقضاء على داء الخيطيات اللمفاوية (2000 – 2014)

هوغو جيم تيرنر، أليسون A بيتيس، وريان ك تشو، ديبورا A مكفارلاند، باميلا ي هوبر، إريك أ أوتيسين ومارك ح برادلي

موجز

نبذة: الخيطيات اللمفاوية الخلفية(LF) :،تعرف أيضا باسم داء الفيل، و هي مرض مداري مهمل (NTD) مستهدف بغرض القضاء عليه من خلال البرنامج العالمي للقضاء على مرض الخيطيات اللمفاوية الخلفية (جبيلف). بين عام 2000 وعام 2014، قدم البرنامج العالمي 5.6 بليون علاجاً إلى أكثر من 763 مليون شخص. من الضروري تحديث تقديرات الفوائد الصحية والاقتصادية لهذا الإنجاز الضخم بغرض تبرير الموارد والاستثمارات اللازمة للقضاء على هذا المرض.

الأسلوب: قمنا بالجمع بين النماذج المحددة سابقا لتقدير عدد المظاهر السريرية وسنوات العمر المعدلة حسب الإعاقة (المصححة) مستبعدة من ثلاثة مجموعات (تلك المحمية من التقاط العدوى والمصابين بالمرض تحت اكلينيكيا حالت دون إحراز تقدم والمصابين بالأعراض السريرية المخففة). تم تحليل الوفورات الاقتصادية المرتبطة بالوقاية من هذا المرض في سياق النفقات الطبية التي تكبدها المرضى السريريون، أثر فقدان الدخل المحتملة من خلال تكاليف العمل المفقودة ومنعت النظام الصحي للعناية بالأفراد. وتم احتساب تقديرات التكاليف غير المباشرة باستخدام نهج رأس المال البشري. واستخدمت مجموعة من أربع مصادر للأجور بغرض تقدير القيمة السوقية العادلة لوقت العامل الزراعي المصاب بعدوى LF) لضمان الحصول على تقديرات متحفظة باستخدام معدلات الأجور الدنيا.

النتائج: توصلنا إلى توقعات بأنه بسبب السنوات ال 15 الأولى فمن المحتمل وقاية عدد 36 مليون حالة سريرية و175 (116-250) (مصححة) من المرض من خلال البرنامج العالمي. ويقدر أنه بفضل هذه التأثيرات الصحية الملحوظة، فمن الممكن توفير مبلغ 100.5 مليون دولار أمريكي على مدى عمر المجموعات المستفيدة من البرنامج. المجموع الإجمالي الناتج من مجموع النفقات الطبية التي تكبدها المصابون بالمرض (3 مليار دولار أمريكي)، تقديرات خسارة الدخل المحتملة (مبلغ 94 مليار دولار أمريكي)، وتبلغ تكلفته على النظام الصحي (مبلغ 3.5 مليار دولار أمريكي) من المتوقع إمكان توفيرها. خضعت النتائج إلى تحليل الحساسية وكانت أكثر حساسية للنسبة المئوية المفترضة لساعات العمل المفقودة لأولئك الذين يعانون من المرض المزمن (تغيير الفائدة الاقتصادية الكلية بين 69.30 مليار – 150.7 مليار دولار أمريكي)

الاستنتاج: رغم محدودية أي تحليل من هذا القبيل، إلا أن هذه الدراسة تحدد الفوائد الصحية والاقتصادية الجمّة التي نجمت عن السنوات الخمس عشرة الأولى من تطبيق البرنامج العالمي، كما أنها تسلط الضوء على قيمة وأهمية مواصلة الاستثمار في البرنامج العالمي للقضاء على داء الخيطيات اللمفاوية

Translated from English version into Arabic by Lamya, through

消除淋巴丝虫病全球计划（(2000–2014)的健康和经济效益

Hugo C Turner, Alison A Bettis, Brian K Chu, Deborah A McFarland, Pamela J Hooper, Eric A Ottesen and Mark H Bradley

摘要

引言：淋巴丝虫病，也被称为象皮病，是一种被忽视的热带病，计划通过消除淋巴丝虫病全球计划（GPELF）消除该病。2000-2014年间，GPELF针对超过7.63亿人已发放了56亿剂治疗。更新估计取得的健康和经济效益的伟大成就对于证明消除淋巴丝虫所需资源和投资的合理性是有深远意义的。

方法：我们结合前期建立的模型来估算临床病例数和伤残调整寿命年（DALY），同时尽量避免从三个利益群体（包括通过感染获得保护的，受阻的亚临床发病和得到缓解的临床病例群体）获益。在淋巴丝虫临床

病例所产生预防医疗费用，丧失劳动力导致的潜在收入损失和医疗体系护理感染个体的预防成本三者背景下，分析疾病控制相关的经济节约。用人力资本法估算间接成本。采用4个工资来源组合法估算1个感染淋巴丝虫的农业工人的当时公平市场价格（为确保保守估计，采用了最低工资标准）。
结果：我们预计 GPELF 的第一个 15 年将有效避免 3 600 万临床病例和 175(116-250)万伤残寿命调整年的发生。据估计，由于这种显著的健康影响，受益人群中有 1 005 亿美元潜在损失得到保留。这个总量包括淋巴丝虫病患者的医疗费用 30 亿，潜在收入损失 940 亿，医疗卫生体系的成本 35 亿。该结果经过敏感性分析，认为对于那些患有慢性疾病的假定工作时间损失是最敏感的，总经济效益为 69.30-150.7 亿美元。
结论：尽管任何此类的分析都存在缺陷，本研究确定了 GPELF 的第一个 15 年所带来的实质性的健康和经济效益，并且进一步表明了继续投资消除淋巴丝虫病全球计划的价值和重要性。

Translated from English version into Chinese by Feng Xin-Yu, edited by Yang Pin, through

CDC CHINESE CENTER FOR DISEASE CONTROL AND PREVENTION
NATIONAL INSTITUTE OF PARASITIC DISEASES

Avantages sanitaires et économiques du programme mondial pour l'élimination de la filariose lymphatique (2000-2014)

Hugo C Turner, Alison A Bettis, Brian K Chu, Deborah A McFarland, Pamela J Hooper, Eric A Ottesen et Mark H Bradley

Résumé

Contexte : La filariose lymphatique, également connue sous le nom d'éléphantiasis, est une maladie tropicale négligée dont l'élimination constitue le but du Programme mondial de l'OMS pour l'élimination de la filariose lymphatique (GPELF). Entre 2000 et 2014, 5,6 milliards de traitements ont été administrés à plus de 763 millions de personnes dans le cadre du GPELF. Un bilan sanitaire et économique de cet importante action s'impose pour justifier les ressources et les investissements nécessaires pour éliminer la filariose lymphatique.
Méthode : Nous avons combiné des modèles déjà établis afin d'estimer le nombre de manifestations cliniques et d'années de vie corrigées du facteur invalidité (AVCI/DALY) évitées dans trois cohortes bénéficiaires (sujets protégés contre l'infection, sujets présentant une infection sous-morbide dont la progression est évitée, sujets infectés dont les symptômes cliniques ont été allégés). Les économies associées à cette prévention de la maladie ont été analysées dans le contexte des dépenses médicales évitées pour les patients présentant une filariose clinique, la perte potentielle de revenus due aux heures de travail perdues et les frais des soins aux sujets atteints évités pour le système de santé publique. Les estimations des coûts indirects ont été calculées selon l'approche du capital humain. Une combinaison de quatre sources de salaires a été utilisées pour estimer la juste valeur de marché du temps de travail d'un ouvrier agricole infecté par la filariose lymphatique (afin d'obtenir une estimation conservatrice, nous avons utilisé la valeur de salaire la plus basse).
Résultats : Selon nos projections, les 15 premières années du GPELF permettront potentiellement d'éviter 36 millions de cas cliniques et 175 (116-250) millions d'AVCI. Nous avons estimé que grâce à cet impact sanitaire notable, 100,5 milliards de dollars US pourraient être économisés sur la durée de vie des cohortes bénéficiaires si l'on additionne les dépenses médicales des patients atteints de filariose lymphatique (3 milliards de dollars), les pertes de revenus potentielles (94 milliards) et le coût pour le système de santé (3,5 milliards) qui pourraient être évités. Les résultats ont été soumis à une analyse de sensibilité et se sont montrés surtout sensibles au pourcentage

supposé d'heures de travail perdues par les patients souffrant d'une atteinte chronique (avec une variation du bénéfice économique total entre 69,30 et 150,7 milliards de dollars).

Conclusions : Malgré les limites inhérentes à ce type d'analyse, notre étude identifie des bénéfices sanitaires et économiques substantiels pour les 15 premières années du GPELF et met en lumière l'intérêt et l'importance de maintenir les investissements dans ce programme.

Translated from English version into French by Suzanne Assenat, through

TRANSLATORS WITHOUT BORDERS

Здоровья и экономической выгоды Глобальной программы по ликвидации лимфатического филяриатоз (2000-2014)

Хьюго С Тернер, Алисон А Беттис, Брайан К. Чу, Дебора А Макфарлэнд, Памела Дж Хупер, Эрик А Оттесен и Марк Н Брэдли

Аннотация

Цель: лимфатической филяриоз (ЛФ), известный также как слоновая болезнь, является тропическим заболеванием, которым не уделяют должного внимания (ЗТЗ), ориентированные на устранение через Глобальной программы по ликвидации ЛФ (ГПЛЛФ). В период с 2000 по 2014 г. ГПЛЛФ проводила 5,6 млрд лечения свыше 763 млн человек. Обновление предположительных лечебных и экономических эффектов от таких значительных достижений играет важную роль в обосновании ресурсов и инвестиций, необходимых для устранения ЛФ.

Метод: Мы объединили установленные ранее модели для оценки количества клинических проявлений и количество утраченных лет жизни с поправкой на длительность инвалидизации (КУЛЖПДИ) отводили из трех полезных когорт (те, которые защищены от инфицирования, те которые с субклинической заболеваемости препрятствующее развития и тех, с клинической картиной заболевания в облегченной форме). Экономическую сбережению, связанные с этой профилактики заболеваний затем анализировали в контексте предотвращенных медицинских расходов, понесенных клинических пациентов по ЛФ, потенциальную потерю доходов за счет потерь рабочей силы, а также предотвратить затраты системы здравоохранения по заботе о пострадавших лиц. Оценки непрямых затрат были рассчитаны с использованием подхода, трудового капитала. Комбинация из четырех источников заработной платы была использована для оценки справедливой рыночной стоимости времени для работника сельского хозяйства с ЛФ-инфекцией (чтобы обеспечить консервативную оценку, использованы наименьшее значение заработной платы).

Результаты: Мы прогнозировали, что из-за первые 15 лет ГПЛЛФ потенциально будет предотвращено 36 миллионов клинических случаев и 175 (116-250) миллионов КУЛЖПДИ. Было подсчитано, что из-за этого заметного влияния на здоровье, 100.5 миллиард долларов США потенциально сохранены в течение жизни контингента населения. Эта сумма является результатом суммирования медицинских расходов, понесенных пациентов ЛФ (3 миллиарда долларов США), потенциальные потери дохода (94 млрд долларов США), а также расходы на систему здравоохранения (3,5 млрд долларов США), что согласно прогнозам, будет

предотвращено. Полученные результаты были подвергнуты анализу чувствительности и были наиболее чувствительны к предполагаемому проценту рабочих часов, потерянных для тех, кто страдает от хронических болезней (изменения общей экономической выгоды между 69.30-150.7 млрд долларов США).

Выводы: Несмотря на ограничения любого такого анализа, данное исследование выявляет значительное лечебные и экономические эффекты, которые привели от первых 15 лет ГПЛЛФ, и это подчеркивает ценность и важность продолжения инвестиций в ГПЛЛФ.

Translated from English version into Russian by Turdimurot Rakhmonov, through

Beneficios económicos y sanitarios del programa global para eliminar la filiariasis linfática (2000-2014)

Hugo C Turner, Alison A Bettis, Brian K Chu, Deborah A McFarland, Pamela J Hooper, Eric A Ottesenand Mark H Bradley

Resumen

Antecedentes: la filiariasis linfática (LF), también conocida como elefantiasis, es una enfermedad tropical desatendida (NTD) que tiene como meta su eliminación a través del Programa global para eliminar la LF (GPELF). Entre los años 2000 y 2014, el GPELF ha implementado 5600 millones de tratamientos a más de 763 millones de personas. La actualización de los beneficios sanitarios y económicos estimados en este logro significativo es importante para justificar los recursos y la inversión necesarios para eliminar la LF.

Método: Hemos combinado los modelos establecidos anteriormente para estimar la cantidad de manifestaciones clínicas y los años de vida ajustados por discapacidad (DALY) que se derivan de tres cohortes con beneficios (personas protegidas de contraer la infección, personas con la enfermedad en fase subclínica en las que se previene el avance y personas con enfermedad en fase clínica aliviada). Los ahorros económicos asociados con la prevención de esta enfermedad se analizaron luego en el contexto de los gastos médicos que se evitaron en los pacientes con LF en fase clínica, la posible pérdida de ingreso por la pérdida de días de trabajo y los costes evitados al sistema de salud por la atención de las personas afectadas. Las estimaciones de costes indirectos se calcularon mediante el concepto de capital humano. Se empleó una combinación de cuatro fuentes de salario para estimar el valor de mercado justo respecto del tiempo relacionado con un trabajador agrícola infectado con LF (para asegurar una estimación conservadora, se empleó el valor de salario más bajo).

Resultados: hemos proyectado que debido a los primeros 15 años del GPELF, se desviarán posiblemente 36 millones de casos clínicos y 175 (116 a 250) millones de DALY. Se estimó que debido a este impacto notable en la salud, posiblemente se ahorrarán cien mil millones y medio de dólares estadounidenses a lo largo del período de vida de los cohortes con beneficios. Este monto total resulta de sumar los gastos médicos requeridos por los pacientes con LF (3000 millones de dólares estadounidenses), pérdida de ingreso potencial (94 mil millones de dólares estadounidenses) y costes para el sistema de salud (3500 millones de dólares estadounidenses) cuya prevención se proyectó. Los resultados están sujetos a un análisis de sensibilidad, donde los más sensibles se reflejan en el porcentaje calculado de horas de trabajo perdidas en personas que sufren de una enfermedad crónica (lo cual modifica el beneficio económico total entre 69.300 y 150.700 millones de dólares estadounidenses).

Conclusiones: a pesar de las limitaciones de cualquiera de estos análisis, en este estudio se identifican los beneficios económicos y sanitarios esenciales que se derivan de la aplicación de los primeros 15 años del GPELF, lo que pone de relieve el valor y la importancia de continuar con la inversión en dicho programa.

Translated from English version into Spanish by Silvia Sassone, through

TRANSLATORS
WITHOUT BORDERS
