# Supplementary material for: The health and economic benefits of the global programme to eliminate lymphatic filariasis (2000–2014)
Source: Infect Dis Poverty. 2016 May 24;5:54. doi: 10.1186/s40249-016-0147-4 (PMC4937583; doi:10.1186/s40249-016-0147-4)
Supplement: Additional file 2: Table S1. — The breakdown of where individuals receive treatment. Table S2. Regional specific patient medical expenses and health system costs. Table S3. DALYs averted stratified by WHO region. Table S4. Economic benefits for individuals and the health systems stratified by the WHO regions. Table S5. Impact of the sensitivity analysis on the projected health benefits of the GPELF (2000–2014). Table S6. Impact of the sensitivity analysis on the projected economic benefits of the GPELF (2000–2014). (DOCX 28 kb) [file 40249_2016_147_MOESM2_ESM.docx]

| **Supporting Table S1: The breakdown of where individuals receive treatment** | | | |
| --- | --- | --- | --- |
| **Parameter** | **Public** **health facility** | **Private** **health facility** | **Self-treat/traditional healers** |
| **Acute (ADL) treatment breakdown** | 25% (India: 55.5%) | 5% (India: 34.5%) | 70% (India: 10%) |
| **Chronic treatment breakdown** | 15% (India: 55.5%) | 5% (India: 34.5%) | 80% (India: 10%) |
| *Based on [*[*1*](#_ENREF_1)*], though updated where appropriate. ADL:* *acute adenolymphangitis* | | | |

| **Supporting Table S2: Regional specific patient medical expenses and health system costs** | | | | |
| --- | --- | --- | --- | --- |
| **WHO region** | **Patient medical expenses per year - Hydrocele** | **Patient medical expenses per year - Lymphedema** | **Patient medical expenses per ADL episode** | **Health system cost per visit** |
| AMRO | $2.24 | $3.36 | $1.56 | $2.12 |
| AFRO | $0.80 | $1.20 | $0.67 | $1.89 |
| EMRO | $0.60 | $0.90 | $0.58 | $5.41 |
| WPRO | $1.40 | $2.10 | $1.18 | $3.98 |
| SEARO | $0.60 | $0.90 | $1.36 | $2.59 |
| **Average** | **$0.70** | **$1.05** | **$1.18** | **$2.46** |
| *For countries where no data was available, the lowest value within the same region was used as a proxy.*  *AMRO: Region of the Americas, AFRO: African Region, EMRO: Eastern Mediterranean Region, WPRO: Western Pacific Region, SEARO: South-East Asia Region. ADL:acute adenolymphangitis. Costs are expressed in US$ 2014 prices. Values shown are weighted averages (based on the benefit cohort population size in each country).* | | | | |

| **Supporting Table S3: DALYs averted stratified by WHO region** | | | |
| --- | --- | --- | --- |
| **WHO region** | **DALYs averted *(millions)**** |  | |
| AMRO | 2.1 (1.413.0) | |  |
| AFRO | 34.0 (22.6-48.5) | |  |
| EMRO | 0.8 (0.6-1.2) | |  |
| WPRO | 6.4 (4.3-9.2) | |  |
| SEARO | 131.8 (87.5-188.1) | |  |
| **Total** | **175.2 (116.3-250.0)** | |  |

*AMRO: Region of the Americas, AFRO: African Region, EMRO: Eastern Mediterranean Region, WPRO: Western Pacific Region, SEARO: South-East Asia Region.*

* *Range based on the 95% uncertainty interval of the disability weight (Table 6).*

| **Supporting Table S4: Economic benefits for individuals and the health systems stratified by the WHO regions** | | | | |
| --- | --- | --- | --- | --- |
| **WHO region** | **Direct costs for individuals prevented - medical expenses** *(millions)* | **Indirect costs for individuals prevented - lost wages** *(millions)* | **Direct costs for the health system** **prevented** *(millions)* | **Total costs prevented** *(billions)* |
| AMRO | $41 | $3,726 | $14 | $3.88 |
| AFRO | $289 | $11,372 | $194 | $11.9 |
| EMRO | $6 | $934 | $13 | $1 |
| WPRO | $89 | $9,545 | $69 | $9.7 |
| SEARO | $2,514 | $68,419 | $3,250 | $74.2 |
| **Total** | **$2,938** | **$93,996** | **$3,540** | **$100.5** |
| *AMRO: Region of the Americas, AFRO: African Region, EMRO: Eastern Mediterranean Region, WPRO: Western Pacific Region, SEARO: South-East Asia Region. Costs are expressed in US$ 2014 prices.* | | | | |

| **Supporting Table S5: Impact of the sensitivity analysis on the projected health benefits of the GPELF (2000-2014).** | | | | | | |
| --- | --- | --- | --- | --- | --- | --- |
|  | **Years of chronic disease averted** | | **Number of acute (ADL) episodes averted** | | **DALYs averted** | |
|  | Lower  Bound | Upper bound | Lower  Bound | Upper bound | Lower  Bound | Upper bound |
| ***Disease Progression & Incidence Rates*** |  |  |  |  |  |  |
| Percentage of clinical patients who experience ADL episodes per year | - | - | -17% | 11% | - | - |
| Frequency of ADL episodes for clinical patients (in absence of MDA) | - | - | -100% | 141% | - | - |
| Disability weight |  |  |  |  | -34% | 43% |
| Mean age of benefit cohorts |  | -18% |  | -19% |  | -18% |
| ***Impact of Treatment*** |  |  |  |  |  |  |
| The reduction in transmission experienced by the treated population | -17% | - | -13% | - | -17% |  |
| Reduction in the frequency of ADL episodes by MDA |  |  | -15% | 16% | - | - |
| Percentage of chronic disease alleviated by MDA | -7% | 38% | -6% | 27% | -7% | 37% |
| *The lower and upper bound parameter ranges are shown in Table 6.* |  |  |  |  |  |  |

|  |  | | | |  | | |  | | |  | | |  |
| --- | --- | --- | --- | --- | --- | --- | --- | --- | --- | --- | --- | --- | --- | --- |
| **Supporting Table S6: Impact of the sensitivity analysis on the projected economic benefits of the GPELF (2000-2014).** | | | | | | | | | | | | | | |
|  | **Direct costs for individuals prevented - medical expenses** | | | **Indirect costs for individuals prevented - lost wages** | | | **Direct costs for the health system** **prevented** | | | **Total costs prevented** | | | | |
| **Parameter** | Lower  Bound | Upper  bound | | Lower  Bound | | Upper  bound | Lower  Bound | | Upper  bound | Lower  Bound | | Upper bound | | |
| ***Disease Progression & Incidence Rates*** |  |  | |  | |  |  | |  |  | |  | | |
| Percentage of clinical patients who experience ADL per year | -13% | 8% | | -3% | | 2% | -8% | | 5% | -3% | | 2% | | |
| Frequency of ADL episodes for clinical patients (in absence of MDA) | -81% | 112% | | -16% | | 22% | -52% | | 71% | -19% | | 27% | | |
| Average duration of an ADL episode | - | - | | -12% | | 20% | - | | - | -11% | | 18% | | |
| Mean age of benefit cohorts | - | -10% | | - | | -10% |  | | -10% | - | | -10% | | |
| ***Patient Medical Expenses and Treatment Seeking Behaviour*** |  |  | |  | |  |  | |  |  | |  | | |
| Percentage of patients with ADL seeking treatment per episode | - | 12% | | - | | - | - | | 19% | - | | 1% | | |
| Percentage of chronic disease patients seeking treatment | -9% | 50% | | - | | - | -6% | | 34% | -0.48% | | 3% |  |  |
| Average patient medical expenses per ADL episode | -16% | 16% | | - | | - |  | |  | -0.48% | | 0.48% | | |
| Average patient medical expenses for chronic disease per year | -5% | 6% | | - | | - | - | | - | -0.15% | | 0.18% | | |
| ***Lost Productivity & Wages*** |  |  | |  | |  |  | |  |  | |  | | |
| Work days per year | - | - | | -11% | | 18% | - | | - | -10% | | 17% | | |
| Percentage of work hours lost per day during an ADL episode | - | - | | -5% | | 4% | - | | - | -5% | | 4% | | |
| Percentage of work hours lost due to chronic disease | - | - | | -31% | | 50% | - | | - | -31% | | 50% | | |
| ***Discounting*** |  |  | |  | |  |  | |  |  | |  | | |
| Discount rate | 46% | -23% | | 48% | | -23% | 44% | | -22% | 47% | | -23% | | |
| ***Impact of Treatment*** |  |  | |  | |  |  | |  |  | |  | | |
| The reduction in transmission experienced by the treated population | -14% | - | | -16% | | - | -15% | | - | -16% | | - | | |
| Reduction in the frequency of ADL episodes by MDA | -13% | 14% | | -3% | | 3% | -8% | | 9% | -3% | | 3% | | |
| Percentage of chronic disease alleviated by MDA | -6% | 31% | | -7% | | 40% | -7% | | 35% | -7% | | 39% | | |
| *The lower and upper bound parameter ranges are shown in Table 6.* | | | |  | |  |  | |  |  | |  | | |
|  |  | |  |  | |  |  | |  |  | |  | | |

**Reference**

1. Chu BK, Hooper PJ, Bradley MH, McFarland DA, Ottesen EA: **The economic benefits resulting from the first 8 years of the Global Programme to Eliminate Lymphatic Filariasis (2000-2007)**. *PLoS Negl Trop Dis* 2010, **4**(6):e708.
